# Evolution of the Alx homeobox gene family: parallel retention and independent loss of the vertebrate *Alx3* gene

**DOI:** 10.1111/j.1525-142X.2011.00489.x

**Published:** 2011-07

**Authors:** Imelda M McGonnell, Anthony Graham, Joanna Richardson, Jennifer L Fish, Michael J Depew, Chris T Dee, Peter WH Holland, Tokiharu Takahashi

**Affiliations:** a Reproduction and Development, The Royal Veterinary CollegeRoyal College Street, London NW1 0TU, UK; b MRC Centre for Developmental Neurobiology, King's College London, New Hunt's HouseGuy's Hospital Campus, London SE1 1UL, UK; c Department of Craniofacial Development, King's College London, Guy's HospitalLondon Bridge, London SE1 9RT, UK; d Department of Orthopaedic Surgery, University of California San Francisco2550 24th Street, SFGH Bldg 9, San Francisco, CA 94110, USA; e Faculty of Life Sciences, The University of Manchester, Michael Smith BuildingOxford Road, Manchester M13 9PT, UK; f Department of Zoology, University of OxfordTinbergen Building, South Parks Road, Oxford OX1 3PS, UK

## Abstract

**SUMMARY:**

The Alx gene family is implicated in craniofacial development and comprises two to four homeobox genes in each vertebrate genome analyzed. Using phylogenetics and comparative genomics, we show that the common ancestor of jawed vertebrates had three Alx genes descendent from the two-round genome duplications (*Alx1, Alx3, Alx4*), compared with a single amphioxus gene. Later in evolution one of the paralogues, *Alx3*, was lost independently from at least three different vertebrate lineages, whereas *Alx1* and *Alx4* were consistently retained. Comparison of spatial gene expression patterns reveals that the three mouse genes have equivalent craniofacial expression to the two chick and frog genes, suggesting that redundancy compensated for gene loss. We suggest that multiple independent loss of one Alx gene was predisposed by extensive and persistent overlap in gene expression between Alx paralogues. Even so, it is unclear whether it was coincidence or evolutionary bias that resulted in the same Alx gene being lost on each occasion, rather than different members of the gene family.

## INTRODUCTION

Homeobox genes are characterized by possession of a particular DNA sequence encoding a recognizable protein domain (Bürglin 1994). More than 200 homeobox genes have been identified in the human genome which can be categorized into 11 classes and around 100 gene families (Holland et al. 2007). The two major classes of homeobox gene are the ANTP and PRD classes, each of which includes many genes implicated in developmental patterning or cellular differentiation (Galliot et al. 1999; Nam and Nei 2005; Ryan et al. 2006;). The ANTP class includes Hox genes, ParaHox genes, NK genes, plus the Emx, Dlx, En, and Evx gene families among others. The PRD class includes other well-known developmental genes including Otx, Otp, Gsc, Pax2/5/8, Pax3/7, Pax4/6, Pitx, and Dmbx.

The Alx homeobox gene family belongs to the PRD class. Humans and mice possess three Alx homeobox genes, namely *Alx1, Alx3*, and *Alx4*. The *Alx1* gene was initially named *Cart1*, but the nomenclature of this gene has been revised to recognize its paralogous relationship. Originally, the Alx genes derived their name from a *Drosophila* gene, *aristaless*, (*a*rista*l*ess homeobo*x*) (Qu et al. 1997a), although the sequence similarity is not high. Indeed, there are several distinct groups of vertebrate genes, notably the Arx and Phox (Arix) gene families that show some similarity to *aristaless* (Beverdam and Meijlink 2001). It was shown later that *aristaless* itself does not belong to the Alx gene family, and that the *Drosophila* ortholog of this family has been secondarily lost in evolution (Ryan et al. 2006). The Alx gene family is certainly ancient, and single representatives have been reported from the sea anemone *Nematostella* (Ryan et al. 2006) and the sea urchins *Strongylocentrotus* and *Lytechinus* (Ettensohn et al. 2003) although the terminology *Alx1* for the latter genes do not reflect one-to-one orthology with vertebrate *Alx1*.

The vertebrate Alx genes have a variety of important developmental roles, being implicated in neural tube closure (Zhao et al. 1996), limb development (Qu et al. 1997b), and most especially aspects of craniofacial development. The three mouse Alx genes show similar developmental expression patterns in the cranial regions of embryo. From early stages (E8.5–E9.5), all three are prominently expressed in frontonasal mesenchyme, and later in the first and second pharyngeal arches (Zhao et al. 1996; Qu et al. 1997a; ten Berge et al. 1998; Beverdam and Meijlink 2001;). Furthermore, gene targeting studies have shown the three Alx genes to have critical roles in craniofacial development in mice (Zhao et al. 1996; Qu et al. 1997b; Beverdam et al. 2001;). In humans, mutations in each of the three Alx genes have been reported to be associated with congenital craniofacial malformation (Wu et al. 2000; Wuyts et al. 2000; Mavrogiannis et al. 2001; Twigg et al. 2009; Uz et al. 2010;), which in each case are closely related to the phenotypes of the mouse mutants.

Although the developmental roles of Alx genes and their molecular interactions with each other have been well studied in mice and humans (Qu et al. 1999; Beverdam et al. 2001;), there remain large gaps in our understanding of the Alx gene family. In particular the evolution of this gene family within the chordates, and, more specifically, the vertebrates has not been scrutinized. Here we report the identification of Alx homeobox genes from amphioxus and from major classes of vertebrates. We have compared embryonic expression patterns between species, and related this to comparative genomic analyses in which we uncover an interesting evolutionary feature: repeated independent loss of the *Alx3* gene.

## MATERIALS AND METHODS

### Cloning of Alx cDNAs

A *Branchiostoma floridae* embryonic cDNA library, kindly provided by Dr. J. Langeland, was screened using an *AmphiDmbx* probe (Takahashi and Holland 2004) under reduced stringency conditions. Two independent clones both containing a full coding region of *AmphiAlx* gene were isolated and sequenced (GenBank: JF460798, JF460799). *Xenopus tropicalis Alx1* and *Alx4* cDNAs (GenBank: JF460800, JF460801) were isolated by RT-PCR amplification from mRNA of stage 35 embryos. Primers used were based on genomic sequence from the Joint Genome Institute draft genome sequence (http://genome.jgi-psf.org/Xentr4/Xentr4.home.html). *Gallus gallus Alx1* and *Alx4* genes were cloned by RT-PCR from chick embryo mRNA. Primers were based on predicted coding sequence.

### Phylogenetic analysis

Amino acid sequences of Alx homeodomain proteins from human and mouse were retrieved from NCBI Entrez Gene as follows: human Alx1 (NP_008913.2), Alx3 (NP_006483.2), Alx4 (NP_068745.2); mouse Alx1 (NP_766141.1), Alx3 (NP_031467.1), Alx4 (NP_031468.1). To identify chicken (*G. gallus*) and zebrafish (*Danio rerio*) sequences, we used reciprocal blast searching with human Alx proteins. Gene family members identified, with nomenclature defined after phylogenetic analysis, were: chicken Alx1 (XP_425445.2) and Alx4 (NP_989493.1); zebrafish Alx1 (NP_001038539.1), Alx3 (XP_695330.1), Alx4a (XP_001340966.1), and Alx4b (NP_001082826.1).

For lizard (*Anolis carolinensis*) and frog (*X. tropicalis*) sequences, tblastn searches using human Alx proteins against whole genome sequence in GenBank (lizard) or DOE Joint Genome Institute (frog) were conducted. *Anolis* sequences deduced were: Alx1 (AAWZ01006799.1–AAWZ01006800.1) and Alx4 (AAWZ01008092.1–AAWZ01008095.1). In *Xenopus*, Alx1 (protein model 196810) and a partial Alx4 (protein 173595) were readily identified in genomic sequence. However, part of the genomic region corresponding to the carboxyl-terminal end of the Alx4 protein is missing in the assembly around this gene, so we cloned the 3′-end of a cDNA by RT-PCR (GenBank: JF460801). We also predicted a further 14 amino acids at the N-terminus from scaffold_281|792714|792755. Echinoderm Alx protein sequences from *Strongylocentrotus purpuratus, Paracentrotus lividus*, and *Lytechinus variegatus* were used as outgroups for the analysis: StrpuAlx (NP_999809.1, originally reported as Alx1) (Ettensohn et al. 2003), LytvaAlx (AAP34699.1, originally reported as Alx1) (Ettensohn et al. 2003), and ParliAlx (ABG00197.1) (Rottinger et al. 2004).

The Alx protein sequences from all 10 species were aligned using ClustalW implemented in the BioEdit software package. The sequences were then manually trimmed of all sites that were not unambiguously aligned. For maximum-likelihood (ML) trees ProtTest 1.4 (Abascal et al. 2005) was used to determine the best protein substitution model and parameters for use in RAxML (Stamatakis et al. 2008). The JTT model of protein evolution was used with the proportion of invariable sites and gamma parameter estimated from the data, four categories of between-site rate variation; 100 bootstraps were used in the primary ML tree (final ML optimization likelihood: −4870.45565). The PHYLIP software package (http://evolution.genetics.washington.edu/phylip/) was used to create neighbor-joining (NJ) trees using the JTT model of protein evolution with 1000 bootstraps.

For identification of the Alx family genes in cartilaginous fish and agnathans, the genome data of the elephant shark (*Callorhinchus milii*; http://blast.fugu-sg.org/) (Venkatesh et al. 2007) and the sea lamprey (*Petromyzon marinus*, PMAR3; http://pre.ensembl.org/Petromyzon_marinus/Info/Index) were searched using the tblasn program with amphioxus and human Alx proteins. Orthology was tested using reciprocal blast searching to the human genome and by phylogenetic analysis (NJ and ML).

For identification of the duplicated *Alx4* genes in teleosts other than zebrafish, genomic information from following sources was used: *Takifugu rubripes* (JGI v4.0; http://genome.jgi-psf.org/Takru4/Takru4.home.html); *Tetraodon nigroviridis* (http://www.ensembl.org/Tetraodon_nigroviridis/Info/Index); *Oryzias latipes* (http://www.ensembl.org/Oryzias_latipes/Info/Index); *Gasterosteus aculeatus* (http://www.ensembl.org/Gasterosteus_aculeatus/Info/Index).

### Synteny analysis

Analysis of genomic regions was used to test hypotheses of gene loss. Genomic regions around *Alx3* and its neighboring genes in zebrafish, zebra finch, mouse, and human were analyzed and compared with corresponding genomic regions of *Xenopus*, lizard, and chicken. Genome assemblies used were from NCBI (*D. rerio* Zv7, *G. gallus* Build 2.1, *Taeniopygia guttata* Build 1.1, *Mus musculus* Build 37.1, *Homo sapiens* Build 36.3; http://www.ncbi.nlm.nih.gov/mapview/), Joint Genome Institute (*X. tropicalis* genome assembly 4.1; http://genome.jgi-psf.org/Xentr4/Xentr4.home.html), and Broad Institute (*A. carolinensis* draft assembly AnoCar 1.0; http://genome.ucsc.edu/cgi-bin/hgGateway?db=anoCar1) (Kent et al. 2002). For genes that have not been annotated, the identity of genes was deduced by reciprocal blast searching to the human genome.

### Whole mount in situ hybridizations

In situ hybridization of whole mount vertebrate embryos was carried out as described (Thompson et al. 2010). Probes for hybridization were synthesized by in vitro transcription using a DIG RNA Labeling Mix (Roche, Mannheim, Germany), following the supplier's instructions, and detection used alkaline phosphatase-conjugated anti-digoxigenin antibody followed by blue staining using NBT-BCIP (Roche).

## RESULTS

### The amphioxus Alx gene

**Fig. 1:**
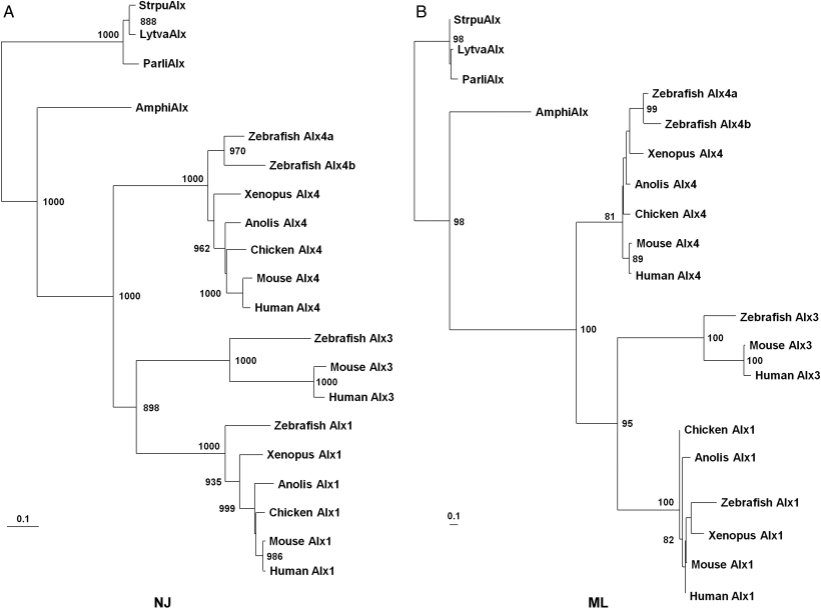
Molecular phylogenetic trees showing the relationships between deuterostome Alx proteins based on neighbor-joining (NJ, A) and maximum-likelihood (ML, B) analyses. Echinoderm Alx proteins were used as outgroups. Branch lengths are proportional to evolutionary distance corrected for multiple substitutions; the scale bar denotes 0.1 underlying amino acid substitutions per site. Figures on branches indicate robustness of each node (>70%), estimated from 1000 bootstrap replicates for NJ (A) and 100 replicates for ML (B). *AmphiAlx* (GenBank: JF460798) is clearly shown to be an ortholog of vertebrate Alx genes by both the NJ (100%) and ML (98%) methods. Furthermore, all extant bony vertebrates share at least two Alx gene duplication events: one that gave rise to the Alx4 and Alx1/3 paralogy groups (100% for both trees), and a second that gave rise to the Alx1 and Alx3 paralogy groups (89.8%, 95%). Further duplication of *Alx4* genes is observed in zebrafish and other teleost fishes (see text).

Two amphioxus cDNA clones (GenBank: JF460798, JF460799) with high sequence similarity to vertebrate Alx genes were isolated. The two clones both include a complete open reading frame and are 98% identical to each other at the amino acid level. Using echinoderm Alx genes as outgroups (Ettensohn et al. 2003), phylogenetic analysis clearly shows that these clones encode the amphioxus ortholog of the vertebrate Alx gene family (*AmphiAlx*, Fig. 1). Our survey of the amphioxus genome (*B. floridae*; http://genome.jgi-psf.org/Brafl1/Brafl1.home.html) by blast revealed the same Alx gene, also identified by Takatori et al. (2008), suggesting that *AmphiAlx* is the single, polymorphic, amphioxus ortholog of vertebrate Alx genes. We discount a very similar sequence (proteinID 69456) neighboring the Alx gene (proteinID 119093) in the draft genome sequence as a probable “stutter” assembly artifact, as noted for other cases (Putnam et al. 2008). In any case, a local tandem duplication would not affect our use of the amphioxus gene as an outgroup for vertebrate sequences. In a previous study (Meulemans and Bronner-Fraser 2007), an EST clone (bfne089p08) was identified as amphioxus Alx; this EST corresponds to the 3′-UTR region of the *AmphiAlx* gene reported here. We also attempted to identify an ortholog in urochordates by blast searching the genomes of *Ciona intestinalis* (http://genome.jgi-psf.org/Cioin2/Cioin2.home.html) and *Ciona savignyi* (http://www.broadinstitute.org/annotation/ciona/). No ortholog was detected suggesting that the Alx gene was lost on the lineage leading to *Ciona*.

### Vertebrate Alx homeobox gene family

In order to examine orthology/paralogy relationships between the multiple Alx genes of different vertebrate species, phylogenetic analysis was performed. The single Alx genes of amphioxus and echinoderm species were ideal outgroups for this analysis. NJ (Fig. 1A) and ML (Fig. 1B) analyses, on amino acid sequence alignments, gave equivalent results.

The phylogenetic analysis confirms that all vertebrate Alx genes form a monophyletic group (NJ 100%, ML 100%), derived from a single ancestral gene. The vertebrate Alx proteins clearly divide into three lineages deduced to be three paralogous genes: *Alx1, Alx3*, and *Alx4*. It is also worth noting that the branch lengths for all *Alx3* proteins are longer than those of other Alx proteins, suggesting that *Alx3* genes may have rapidly evolved in comparison with *Alx1* and *Alx4*.

As each of the three genes can be found in at least some species of actinopterygian and sarcopterygian vertebrates, we suggest that the three Alx genes were generated by the two-round whole genome duplications (2R-WGD) that took place in early vertebrate evolution (Dehal and Boore 2005; Holland et al. 2007;). Our analysis places *Alx1* and *Alx3* closer to each other than to *Alx4* (NJ 89.8%, ML 95%). The first genome duplication will have generated two Alx genes, and we suggest that *Alx1* and *Alx3* were generated from one of these in the second genome duplication, whereas the other Alx gene gave rise to *Alx4* and an “*Alx2* ” gene that has not been detected. Subsequently, “*Alx2* ” has been lost from all extant vertebrates examined to date.

For further elucidation of the early evolutionary history of vertebrate Alx genes, we also analyzed the genomic information of basal vertebrates available to date, namely cartilaginous fishes and agnathans. In chondrichthyes, draft genome sequence data from the elephant shark (*C. milii*) was analyzed. Despite its low genome coverage (1.4 ×), the genome sequences include three different sequences closely related to the homeodomain of human Alx proteins (GenBank: AAVX01553552.1, AAVX01326612.1, AAVX 01282984.1) or the carboxyl-terminal OAR domain of Alx proteins (Beverdam and Meijlink 2001) (GenBank: AAVX01514414.1, AAVX01274664.1, AAVX01004849.1). Our phylogenetic analysis using the OAR regions suggests these sequences are orthologs to *Alx1, Alx3*, and *Alx4* of jawed vertebrates (NJ, ML, data not shown). This implies that the three distinct vertebrate Alx genes arose before the divergence of chondrichthyans and osteichthyans, compatible with origin during the 2R-WGD. We cannot deduce when loss of *Alx2* occurred, however, from low coverage genome data. Analysis of draft genome sequence from the sea lamprey (*P. marinus*) revealed two contigs (Ensembl: Contig 7807, 24307) containing homeobox sequences from the Alx family (NJ, ML, data not shown). The lack of a genome assembly precludes further phylogenetic analysis.

Human and mouse have one each of the three Alx genes (*Alx1, Alx3, Alx4*), but quite different complements of genes are found in zebrafish, frog, chick, and lizard. For frog, chick, and lizard, only two genes were identified; phylogenetic analysis clearly shows these are *Alx1* and *Alx4*. In the zebrafish genome, four Alx genes were identified. Our analysis shows that the protein denoted zgc: 162606 (GenBank: NP_001082826) is most closely related to the protein named zebrafish *Alx4* (GenBank: XP_001340966). The implication is that zebrafish has two *Alx4* genes, which we name *Alx4a* (GenBank: XM_001340930) and *Alx4b* (GenBank: NM_001089357). The duplication of *Alx4* genes is also observed in the genomes of *Takifugu* (JGI: 598617, 579380), *Tetraodon* (GenBank: CAG08730, CAF99670), medaka (Ensembl: ENSORLT00000005949, ENSORLT00000008228), and stickleback (Ensembl: ENSGACT00000020637, ENSGACT 00000013078). This suggests that the duplication occurred in the teleost fish lineage after it had diverged from the other vertebrates. This conclusion is further supported by the observation that the two teleost *Alx4* genes are located in duplicated chromosomal regions showing synteny to the human *Alx4* genomic region (data not shown).

### Independent losses of the *Alx3* gene

**Fig. 2:**
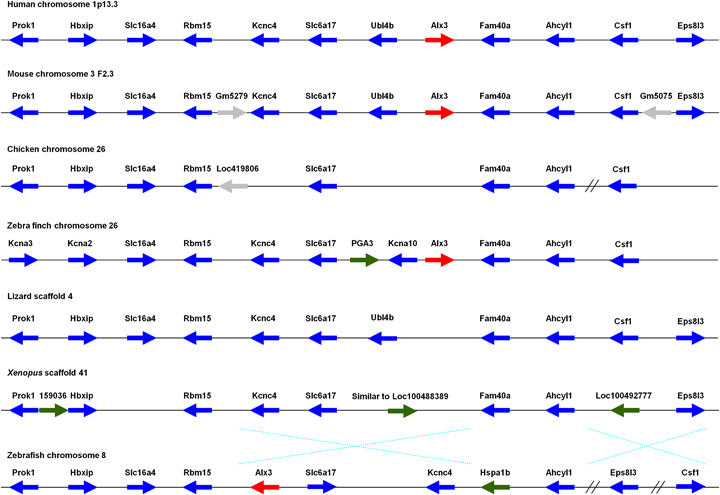
Schematic diagram showing the location of *Alx3* and neighboring genes in syntenic regions of *Homo sapiens, Mus musculus, Gallus gallus, Taeniopygia guttata, Anolis carolinensis, Xenopus tropicalis*, and *Danio rerio*. Arrows indicate genes and their direction of transcription. The blue/black arrows represent loci for which syntenic conservation is confidently identified (zebra finch Kcna genes are denoted by blue arrows because their orthologs in other vertebrates are located immediately downstream of *Prok1*). *Alx3* genes are highlighted with red arrows/gray shadows. The green/gray arrows denote loci for which orthologs are not located in the syntenic region; these are probably inserted secondarily. The gray arrows denote predicted pseudogenes. The dotted crosses indicate inversions within this region in zebrafish genome. The double oblique lines in the zebrafish and chicken genomic regions indicate presence of other genes between the loci shown.

The apparent absence of *Alx3* in frog, chick, and lizard is intriguing. To test whether this reflects a true gene loss, or just incomplete data, we examined genome sequences for these species. Sequence searching using blast failed to identify orthologs of this gene in the draft genomes for any of the three species. More conclusive evidence came from examination of the genomic regions deduced to be syntenic to those harboring the *Alx3* gene in human, mouse, and zebrafish (Fig. 2). In all three cases—frog, chick, and lizard—we found clear syntenic regions containing orthologs of many of the genes surrounding *Alx3* in human, mouse, and zebrafish. Even the gene order is well conserved, apart from an inversion in zebrafish (Fig. 2). However, the *Alx3* gene is clearly absent in frog, chick, and lizard from its expected position between *Slc6a17* and *Fam40a* genes.

We further extended our survey of the *Alx3* genomic region to a larger number of vertebrate species. The *Alx3* gene was found to be present in all mammalian and fish species for which there was adequate genome coverage (not shown). More surprisingly, we found the *Alx3* gene in the zebra finch (*T. guttata*) genome, located in the appropriate syntenic region (Fig. 2).

It is clear, therefore, the *Alx3* gene has been lost in evolution from the genomes of frog (*X. tropicalis)*, lizard (*A. carolinensis*), and chicken (*G. gallus*). The known phylogenetic relationships between species possessing *Alx3* and species lacking *Alx3* reveal that these have been independent losses.

### Consequences of gene loss on Alx gene expression

**Fig. 3:**
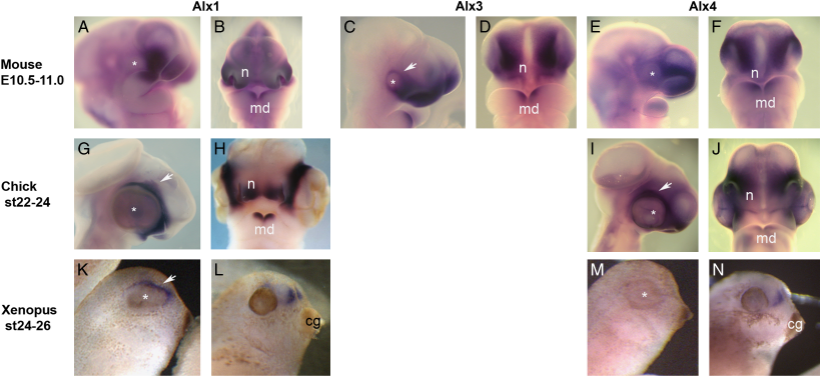
Alx expression in the developing head of mouse, chick, and frog embryos. Expression of mouse, chick, and *Xenopus* Alx genes during craniofacial development was compared using whole mount in situ hybridization. Lateral (A, C, E, G, I, K, L, M, N) and frontal (B, D, F, H, J) views of the developing head are shown. At E10.5-11 in mouse, *Alx1* (A, B), *Alx3* (C, D), and *Alx4* (E, F) are expressed in the mesenchyme of the facial prominences, particularly around the nasal region (n), and at the distal tip of the mandible (md). At this stage, *Alx3* is additionally expressed in the mesenchyme (C, white arrow) encircling the eye. (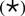). At stages 22–24 of chick development, *Alx1* (G, H) and *Alx4* (I, J) are also expressed in the facial prominences, again with strong expression around the nasal region (n), and at the distal tip of the mandible (md). In the chick, however, both *Alx1* (G) and *Alx4* (I) are expressed in the periocular mesenchyme (white arrows). Periocular expression of *Alx1* is observed in frog embryos at the stage of 24 (K, white arrow). At this stage, *Alx4* is not expressed in frog embryos (M) but later at stage 35, both *Alx1* (L) and *Alx4* (N) are expressed in frontal mesenchyme above the cement gland (cg).

To determine whether the loss of *Alx3* in evolution is associated with differences in expression of *Alx1* and *Alx4*, we compared Alx gene expression in the developing head of mouse, chick, and frog embryos of comparable stages (Fig. 3). This region of the embryo is the major site of expression of Alx genes and displays phenotypic alterations in mice and humans carrying Alx mutations.

Consistent with previous reports, we find expression of mouse *Alx1, Alx3*, and *Alx4* in the craniofacial mesenchyme of embryos at E10.5, with expression domains that largely overlap (Fig. 3, A–F). All three genes are expressed in the forming facial prominences and in the distal tip of the mandible. There is particularly strong expression around the nasal region. At this stage, mouse *Alx3* is expressed in the periocular mesencyme and can be seen to circumscribe the eye (Fig. 3C, white arrow).

The expression of Alx genes in the developing chick head has not been reported previously. We find that expression of chick *Alx1* and *Alx4* is comparable to mouse Alx gene expression (Fig. 3, G–J). At stages 22–24 of development, both *Alx1* and *Alx4* are expressed in the facial prominences and the distal tip of the mandible and again there is strong expression around the nasal region. There is, however, an interesting difference in that chick *Alx1* and *Alx4* are expressed in the periocular mesenchyme encircling the eye in the chick at this stage (Fig. 3, G and I, white arrows). In mouse, this region expresses *Alx3*, but not *Alx1* or *Alx4*.

In *Xenopus*, the *Alx1* gene (Fig. 3K, white arrow) but not *Alx4* (Fig. 3M) is expressed in the mesenchyme encircling the eye at stage 24. Later, at stage 35, expressions of both *Alx1* and *Alx4* are observed in frontal mesenchyme close to the hatching gland (Fig. 3, L and N). The slight difference in expression timing compared with amniotes is possibly due to the existence of the cement gland. Nevertheless, the sum of both spatial and temporal expression patterns of *Xenopus* Alx genes is largely equivalent to that seen for chick and mouse Alx genes.

## DISCUSSION

### Evolution of vertebrate Alx homeobox gene family

**Fig. 4:**
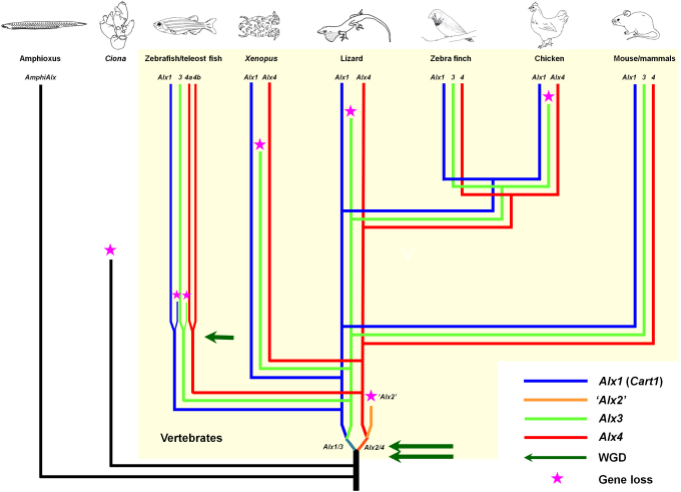
“Gene family tree” of vertebrate Alx homeobox genes in chordate evolution. After the two whole genome duplications (WGD, two green arrows at bottom of diagram) in early vertebrate evolution, four Alx homeobox genes were generated. The “*Alx2*” gene (yellow line) may have been swiftly lost, whereas the *Alx3* genes were lost independently in at least three lineages (shown as the stars). Further modification in the teleost fish lineage followed the third WGD. The diagram depicts the phylogenetic location of each *Alx3* gene loss, but note that the timing of loss within each lineage has not been determined.

The accepted definition of a homeobox “gene family” is that set of genes descendent from a single gene in the common ancestor of bilaterian animals (Holland et al. 2007). In several invertebrates, including the sea anemone *Nematostella* (Ryan et al. 2006), sea urchin (Ettensohn et al. 2003), and amphioxus (Meulemans and Bronner-Fraser 2007; Takatori et al. 2008; and this study), only a single member of the Alx homeobox gene family has been identified. The implication is that the ancestral Alx gene has not duplicated in these lineages. In two *Ciona* species (tunicates), plus the nematode *Caenorhabditis elegans* and the insects *Drosophila, Tribolium*, and *Apis*, no Alx gene has been found. This implies secondary loss of the gene. In contrast, in vertebrates the Alx gene family includes three distinct genes—*Alx1, Alx3*, and *Alx4*—deduced to be descendent from the two WGDs that occurred in early vertebrate evolution (2R-WGD) (Holland et al. 2007; Putnam et al. 2008;) (summarized in Fig. 4). As *Alx1* and *Alx3* are more closely related to each other than either is to *Alx4* (Fig. 1), it seems likely that the first round of genome duplication generated the common ancestor of *Alx1* and *Alx3* genes (*Alx1/3* in Fig. 4) plus the ancestor of *Alx4* gene (*Alx2/4*). The next duplication event gave rise to *Alx1* and *Alx3* genes from the *Alx1/3* gene, and *Alx4* and another Alx gene (“*Alx2* ”) from the other (Fig. 4). This “*Alx2* ” gene has not been identified in any vertebrate so far, and may have been lost soon after the 2R-WGD.

In the teleost fish lineage, a further WGD occurred, subsequent to its divergence from basal actinopterygian fish (Jaillon et al. 2004). This will have generated duplicated *Alx1, Alx3*, and *Alx4* genes, although our genomic analysis of five teleost species (zebrafish, *Tetraodon, Takifugu*, medaka, and stickleback) showed that only the duplicated *Alx4* genes (*Alx4a, Alx4b*) were retained in these species. Duplicated *Alx1* and *Alx3* genes were lost after the third WGD in teleosts.

### The enigma of vertebrate Alx3

The three principal members of the vertebrate Alx gene family (*Alx1, Alx3*, and *Alx4*) were established early in vertebrate evolution, and all were still present in the genome of the common ancestor of Chondrichthyes, Actinopterygii, and Sarcopterygii. However, database searching and analysis of syntenic regions reveal, definitively, that the *Alx3* gene has been lost from the genome of at least three vertebrate species: the amphibian *X. tropicalis*, the squamate reptile *A. carolinensis*, and the bird *G. gallus*. These must all be independent losses as proved by the presence of the *Alx3* gene in zebrafish, zebra finch, and mammals. Thus, the species which lack the *Alx3* gene are on different evolutionary lineages, separated by lineages that possess *Alx3* (Fig. 4).

Why should *Alx3* be lost repeatedly, but not *Alx1* or *Alx4*? One relevant finding is that sequence analysis suggests that *Alx3* and *Alx1* are more closely related to each other than they are to *Alx4*. Consistent with this evolutionary inference, *Alx1* and *Alx3* (but not *Alx4*) have similar roles in neural tube closure (Zhao et al. 1996; Lakhwani et al. 2010;), whereas *Alx4* is the only member involved in the axial patterning of limbs in chicken and mouse (Takahashi et al. 1998). This suggests that losing *Alx3* or *Alx1* could be more easily compensated for than losing *Alx4*. To assess why it is *Alx3*, not *Alx1*, that has been repeatedly lost, it is necessary to consider the expression of these genes.

### Evolution of Alx gene expression and function in vertebrate embryos

The expression and function of mouse Alx family genes have been well studied (Zhao et al. 1996; Qu et al. 1997b, 1999; Beverdam et al. 2001; Lakhwani et al. 2010). All three genes are expressed in cephalic mesenchyme at early stages of mouse development and, consistent with this, mutations in each Alx gene show similar craniofacial phenotypes including defects of the facial bones. Clinical cases involving mutation of any one of the three human genes also display craniofacial deformity (Wu et al. 2000; Wuyts et al. 2000; Mavrogiannis et al. 2001; Twigg et al. 2009; Uz et al. 2010;). Although there are slight differences between phenotypes, and the exact developmental roles are not known, it is clear that each of the Alx genes plays an important role in craniofacial formation in both mouse and human.

We have analyzed the expression of Alx genes during head development in chick and frog embryos and compared expression with Alx genes of mouse. We find that expression is broadly similar between the three species. More specifically, the two Alx genes of chick or frog are expressed in patterns comparable to the sum of the three Alx genes of mouse. Interestingly, we note that chick *Alx1* and *Alx4*, and *Xenopus Alx1*, are expressed in the embryonic periocular mesenchyme; in mouse neither *Alx1* nor *Alx4* shows specific expression in this region. Instead, it is mouse *Alx3* that is expressed in the mesenchyme encircling the eye.

This finding may be relevant to understanding the loss of *Alx3* in evolution. One possibility is that the loss of *Alx3* in chick and frogs was tolerated in evolution because *Alx1* or *Alx4* expression compensates for the Alx role in periocular mesenchyme, and possibly other roles. Whether this is a reflection of pre-existing overlap of function (redundancy) before gene loss, or a subsequent change in gene expression following gene loss, is unclear. The former may seem more logical, but recent data from mouse mutants (Lakhwani et al. 2010) suggests the latter possibility should not be discounted. Although not noted explicitly by the authors, a recent analysis of *Alx3*^−/−^ mutant mice showed a slightly altered expression pattern of *Alx4*, including an apparently novel ring of gene expression close to the eye region (Fig. 3I in Lakhwani et al. 2010). This suggests that Alx genes suppress each other in certain spatial domains, and that after gene loss a degree of functional compensation may occur. If the mechanism whereby *Alx3* suppresses expression of *Alx1* and *Alx4* was established early in vertebrate evolution, then loss of *Alx3* might have been readily compensated by activating expression of Alx paralogous genes. In this scenario no alteration of *cis*-regulatory elements was necessary, implying that *Alx3* might be easily lost in evolution. A possible step toward testing this hypothesis would be to use morpholinos to suppress the action of *Alx3* mRNA in zebrafish embryos.

If loss of *Alx3* can be compensated by paralogous Alx expression, we should also ask why some species retain the *Alx3* gene. One possibility is that Alx3 has evolved new roles in some vertebrates. For example, our phylogenetic trees show longer branch lengths leading to Alx3 proteins, suggesting more rapid evolution of peptide sequence. Mouse and human Alx3 proteins also contain discrete functional domains not possessed by other Alx proteins (Perez-Villamil et al. 2004). We suggest, therefore, that retention of *Alx3* may be explained through the acquisition of new protein domains, interactions, and roles in certain vertebrate lineages.

These changing gene expression patterns, within and between species, together with multiple gene losses and changes to gene family composition, reveal a dynamic picture of Alx gene structure and function through vertebrate evolution. Similarly, craniofacial development is one of the most complicated aspects of vertebrate embryology, and it too has undergone drastic changes in vertebrate evolution. The relationship between the two is not straightforward, but the study of Alx genes has at least provided a glimpse into the complex relation between genome evolution and the developmental basis of craniofacial evolution.
